# Surface Texturing of Si with Periodically Arrayed Oblique Nanopillars to Achieve Antireflection

**DOI:** 10.3390/ma14020380

**Published:** 2021-01-14

**Authors:** Jun-Hyun Kim, Sanghyun You, Chang-Koo Kim

**Affiliations:** 1 Institute of Convergent Chemical Engineering and Technology, SungKyunKwan University, Seobu-ro 2066, Jangan-gu, Suwon 16419, Korea; junhyun0401@gmail.com; 2 Department of Chemical Engineering and Department of Energy Systems Research, Ajou University, Worldcup-ro 206, Yeongtong-gu, Suwon 16499, Korea; you15717@ajou.ac.kr

**Keywords:** surface texturing, oblique nanopillars, slanted plasma etching, light reflection, antireflection

## Abstract

Si surfaces were texturized with periodically arrayed oblique nanopillars using slanted plasma etching, and their optical reflectance was measured. The weighted mean reflectance (*R_w_*) of the nanopillar-arrayed Si substrate decreased monotonically with increasing angles of the nanopillars. This may have resulted from the increase in the aspect ratio of the trenches between the nanopillars at oblique angles due to the shadowing effect. When the aspect ratios of the trenches between the nanopillars at 0° (vertical) and 40° (oblique) were equal, the *R_w_* of the Si substrates arrayed with nanopillars at 40° was lower than that at 0°. This study suggests that surface texturing of Si with oblique nanopillars reduces light reflection compared to using a conventional array of vertical nanopillars.

## 1. Introduction

Silicon (Si)-based photovoltaic devices, such as solar cells, have been studied extensively due to the strong demand for clean and renewable energy. Although there have been technological developments in the photovoltaic industry over the last decades, light reflection from Si surfaces (due to the high refractive index of Si) has been an inherent limitation in the power conversion efficiency of solar cells [1]. Antireflection techniques are used to rectify this issue; they are primarily implemented with either surface coating [2] or surface texturing [3].

Surface coating with antireflective layers is a conventional and simple method for reducing reflectivity loss. Light reflection can be reduced by manipulating the refractive index of the coating layer. However, coating foreign materials onto Si surfaces causes thermal instability due to the different thermal expansion properties of each material [1]. This eventually leads to delamination of the coated layers during thermal cycling. Furthermore, single-layer coatings are effective only for relatively narrow wavelength bands, meaning that complex layer stacks are required to achieve broadband antireflection effects [4].

Reflectivity loss can also be reduced by texturing Si surfaces. Surface texturing methods with various structures, such as rods [5], pyramids [6], tips [7], and cones [8], have been proposed and fabricated. Surface texturing can be performed via either wet [9] or dry [10] etching. Wet etching is a simple method for fabricating surface structures, but it presents less freedom in choosing the substrate (e.g., crystallinity) and requires substantial amounts of chemicals. In addition, it is extremely difficult to precisely control the position and size of the surface structures created during wet etching.

The dry etching method is based on gaseous plasmas; it has some advantages over wet etching. Plasma etching allows for selective and anisotropic etching, and allows the shape of the surface structures to be controlled. In addition, fewer toxic chemicals are used in plasma etching compared to wet etching. Shieh et al. [11] fabricated silicon nanograss with an average diameter of 20 nm using hydrogen plasma etching. They observed a clear decrease in the optical reflectance. Chen et al. [12] performed plasma etching in an Ar/BCl_3_/Cl_2_ mixture to form nanocylinder structures on the surface of Si. They analyzed the optical properties of cylindrical nanostructures with different widths. Pai et al. [5] investigated the aspect-ratio-dependent reflection of silicon nanopillars produced by CF_4_/Ar plasma etching on a Si substrate. They suggested that Si nanopillars with higher aspect ratios cause lower reflectance. Lee et al. [7] achieved Si nanotips with radii ranging from 20 to 100 nm using a two-step dry etching (CF_4_/O_2_ reactive ion etching followed by SF_6_/C_4_F_8_ deep reactive ion etching) process. They studied the effects of the density and aspect ratio of these nanotips on the reflectance.

The light-trapping effect is the main cause for the decreased optical reflectance of textured surfaces [13]. In other words, light is trapped in the trenches (or valleys) between the surface structures (pillars, wires, rods, etc.), leading to a reduction in the light reflection. From this point of view, optical reflectance could be further reduced by texturing a surface with oblique (off-normal to the surface) structures because the trenches between the oblique structures would provide longer paths for light-trapping. Xu et al. [14] fabricated black Si with oblique nanocone arrays through a tilted etching process, and showed that the surface-enhanced Raman scattering of the substrate with oblique nanocones was greatly improved. Yao et al. [15] presented a two-dimensional (2D) Si-nanorod array with oblique indium–tin–oxide (ITO) film using oblique-angle deposition. They demonstrated that 2D Si-nanorod arrays with oblique ITO films had strong optical absorption. However, there have been few reports on antireflective surfaces with oblique structures fabricated through plasma etching.

In this study, Si surfaces with periodically arrayed oblique nanopillars were fabricated via slanted plasma etching, and their optical reflectance was measured. Oblique Si nanopillars were created using a Faraday cage, which can control the angle of ions incident on the substrate surface. A range of angles of the nanopillars from 0° (vertical to the surface) to 60° was utilized to investigate the effect of the nanopillar inclination on the optical properties of the Si surface. The aspect ratios of the vertical and oblique nanopillars were also varied, and their optical reflectance was compared.

## 2. Materials and Methods

**Figure 1:**
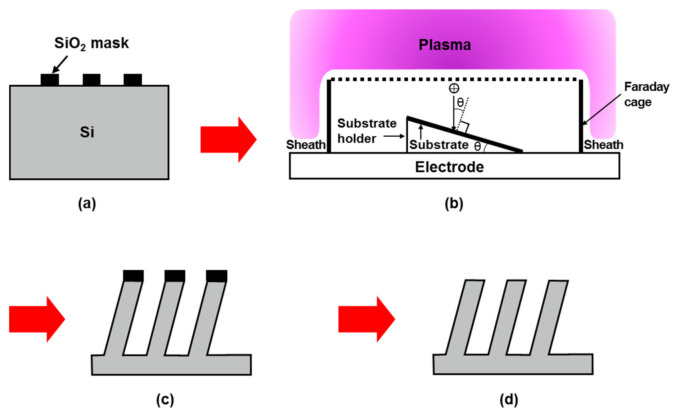
Schematic of the fabrication process of periodically arrayed oblique Si nanopillars: (**a**) Si substrate with hole patterns and a SiO_2_ mask; (**b**) slanted plasma etching using a Faraday cage system; (**c**) oblique etch structures formed after the slanted plasma etching; (**d**) oblique Si nanopillars formed following removal of the SiO_2_ mask.

Figure 1 shows a schematic of the fabrication process for periodically arrayed oblique Si nanopillars. A Si substrate with hole patterns and a SiO_2_ mask was etched via slanted plasma etching. The slanted plasma etching technique is suitable for the fabrication of oblique etched structures over a large area, and can achieve excellent uniformity regarding pattern height and diameter [16]. The slanted plasma etching was performed using a Faraday cage system. A Faraday cage is a closed box made of a conductor, and its top plane is made of a conductive grid. If the cage is in contact with the plasma and the size of the grid’s opening is smaller than the sheath thickness, then a sheath will form along the top plane of the cage. This means that ions generated from the plasma entering the cage will align vertically. When traveling inside the cage, the ions maintain their initial direction because the electric potentials are uniform within the cage. In other words, the initial direction of the ions is not affected by the angle of the substrate holder in the cage. Therefore, the ion-incidence angle (θ), i.e., the angle of the ions incident on the surface of the substrate, can be precisely controlled by varying the angle of the substrate holder. θ is defined as the angle between the ion-incidence direction and the surface normal to the substrate. In this study, after the slanted plasma etching, the SiO_2_ masks on the oblique etch structures were removed using a hydrofluoric (HF) solution (20%) to form oblique Si nanopillars.

**Figure 2:**
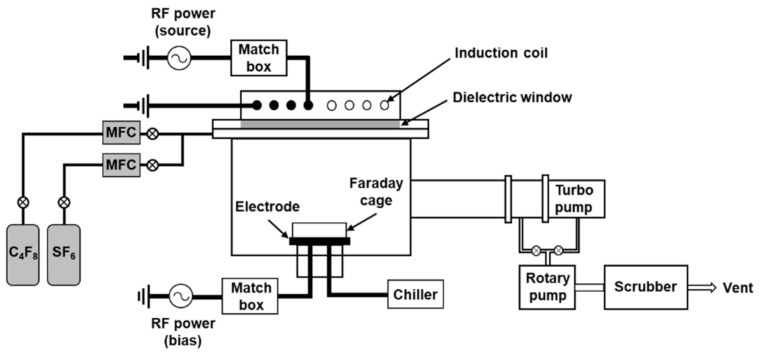
Schematic of an inductively coupled plasma system equipped with a Faraday cage to perform slanted plasma etching.

Slanted plasma etching was performed in an inductively coupled plasma (ICP) system, as shown in Figure 2. The ICP was ignited by applying a 13.56 MHz radio frequency (RF) source power to the induction coil. A separate 13.56 MHz RF bias power was applied to an electrode to independently bias a sample on the electrode. The ICP chamber was equipped with a Faraday cage, which was fixed to the electrode. The grid diameter and pitch of the Faraday cage used in this study were 25 and 229 μm, respectively. The angle of the substrate holder was varied between 0° and 60° to fabricate the oblique Si nanopillars at various angles.

**Figure 3:**
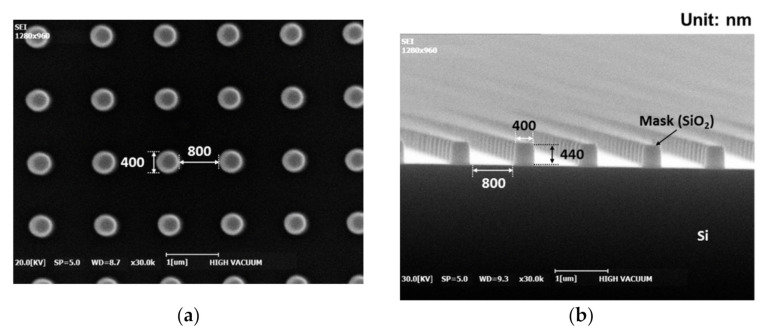
Scanning electron microscopy (SEM) images of the hole patterns on the samples: (**a**) top; (**b**) cross-sectional view.

Periodically arrayed oblique Si nanopillars were fabricated on a p-type Si (100) wafer. Figure 3 shows scanning electron microscopy (SEM) images of the samples. The sample comprised a Si substrate featuring hole patterns with a SiO_2_ mask. The diameter and pitch of the holes were 400 and 1200 nm, respectively. Thus, the holes were spaced 800 nm apart. The height of the hole mask was 440 nm.

Slanted plasma etching was performed through a cyclic process consisting of alternating etching and deposition steps. This cyclic process has been widely used to obtain deep Si etch profiles [17]. In the etching step, SF_6_ plasmas were used by applying 400 W and −100 V as the source power and bias voltage, respectively. The flow rate of SF_6_ was 30 sccm (sccm denotes cubic centimeters per minute at standard temperature and pressure), and the chamber pressure was 10 mTorr (1.3 Pa) during this step. In the deposition step, C_4_F_8_ plasmas were used by applying only a source power of 400 W. The flow rate of C_4_F_8_ was 30 sccm, and the chamber pressure was 30 mTorr (4.0 Pa) during the deposition step. The etching and deposition steps lasted for 21 and 5 s, respectively. The alternating etching and deposition steps were repeated for as many cycles as needed to obtain the desired etch depth.

The heights of the nanopillars (defined as the etch depth in the direction of θ) were fixed to 1500 nm in this study. The number of cycles to obtain 1500-nm-high nanopillars at each angle was as follows: 11 cycles for 0°, 11 cycles for 10°, 11 cycles for 15°, 12 cycles for 20°, 12 cycles for 30°, 14 cycles for 40°, 15 cycles for 50°, and 18 cycles for 60°. The aspect ratios of the nanopillars at 0° and 40° were varied accordingly to compare the effects of the aspect ratio of the trench between the nanopillars at different angles. The aspect ratio of the structure (either nanopillar or trench between the nanopillars) was defined as the height of the structure divided by its width.

When a sample is exposed to fluorocarbon plasmas, thin fluorocarbon films form on its surface [18]. After slanted plasma etching, the residual fluorocarbon films on the nanopillars were removed by ashing them at 500 °C for 1 h. The samples were then dipped in an HF solution (20%) for 150 s to remove the SiO_2_ masks before being rinsed with deionized water.

The etch profiles of the samples were observed using SEM (COXEM, EM-30AX, Daejeon, Korea). The optical properties of the samples were evaluated using the reflectance spectra in the range of 300–800 nm, as obtained by an ultraviolet–visible (UV–Vis) spectrometer (Jasco, V-650, Tokyo, Japan). The UV–Vis spectrometer was equipped with an integrating sphere (Jasco, ISV-722, Tokyo, Japan) with normal incidence (incidence angle to the reflection surface = 0°).

**Figure 4:**
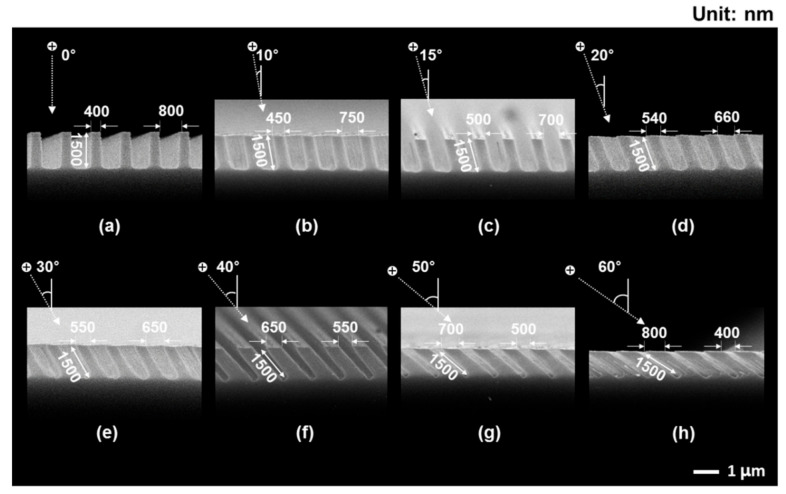
SEM images of Si substrates with periodically arrayed nanopillars at various ion-incidence angles: (**a**) 0°; (**b**) 10°; (**c**) 15°; (**d**) 20°; (**e**) 30°; (**f**) 40°; (**g**) 50°; (**h**) 60°. The heights of the nanopillars were defined as the etch depth in the direction of the ion-incidence angle.

Figure 4 shows SEM images of Si substrates with periodically arrayed nanopillars that were fabricated using slanted plasma etching at θ = 0°, 10°, 15°, 20°, 30°, 40°, 50°, and 60°. For all cases, the heights of the nanopillars (vertical or oblique) were set to be 1500 nm in the direction of θ by manipulating the number of cycles during slanted plasma etching. The Si nanopillars were uniformly arrayed at each angle. The SEM images show that the angles of the oblique Si nanopillars corresponded well to the respective angles of ions incident on each substrate.

## 3. Results and Discussion

**Figure 5:**
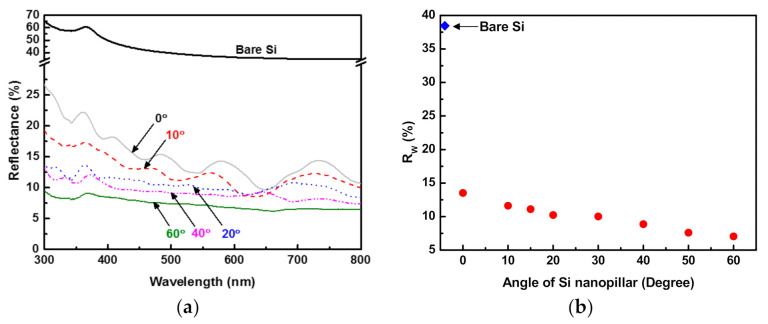
(**a**) Optical reflectance of Si substrates periodically arrayed with nanopillars at various angles; (**b**) weighted mean reflectance of Si substrates with periodically arrayed nanopillars as a function of their angles. In both figures, the values of bare Si are included for comparison.

Figure 5a shows the optical reflectance of Si substrates periodically arrayed with nanopillars at various angles. The reflectance of bare Si was also included for comparison. Bare Si exhibited a reflectance above 40% in the UV–visible range (300–800 nm) and reached as high as 64% at 300 nm. The surfaces of the Si substrates textured with periodically arrayed oblique nanopillars exhibited optical reflectance that was greatly reduced. In order to quantitatively compare the reflectance loss from the bare Si, the weighted mean reflectance (*R_w_*) was calculated using Equation (1), where *R*(*λ*) is the reflectance of the Si surfaces with periodically arrayed oblique nanopillars and *S_AM_*_1.5*G*_ is the air mass (AM) at 1.5 G solar spectral irradiance [19].

(1) $$R_{W}=\frac{\int_{300\;\mathrm{nm}}^{800\;\mathrm{nm}}R\left(\lambda \right)S_{AM\;1.5G}\left(\lambda \right)d\lambda }{\int_{300\;\mathrm{nm}}^{800\;\mathrm{nm}}S_{AM\;1.5G}\left(\lambda \right)d\lambda }$$

Figure 5b shows the *R_w_* values of the Si substrates with periodically arrayed nanopillars as a function of their angles. The *R_w_* for bare Si was 37%, which was significantly higher than those of the substrates with periodically arrayed nanopillars. When the Si surface was textured with vertical nanopillars, its *R_w_* was 13.5%. *R_w_* decreased monotonically with the increase in the angle of the Si nanopillars. The lowest *R_w_* (7.1%) was obtained at 60°, indicating that the reflectance of bare Si was greatly reduced by the formation of periodically arrayed oblique Si nanopillars.

**Figure 6:**
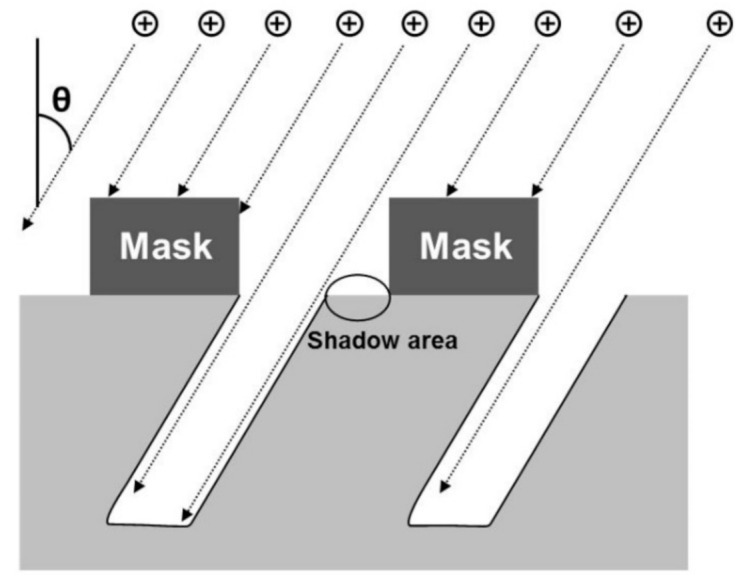
Illustration of the shadowing effect due to the ions incident on the substrate in the oblique direction from the surface normal.

The reduction in the *R_w_* of the nanopillar-arrayed Si substrate with increasing angle of the nanopillars may have resulted from the decreasing width of the trench between the nanopillars due to the so-called shadowing effect [16]. When θ is oblique from the surface normal to the substrate, a mask prohibits ions from arriving at the substrate. This causes a shadow area, as shown in Figure 6. As θ is increased, the shadow area increases, which corresponds to a decrease in the etchable area. Therefore, the width of the trenches between the nanopillars decreases (or the diameters of the nanopillars increase) as the angles of the nanopillars increase. As shown in Figure 4, the diameters of the nanopillars increased from 400 to 800 nm as their angles increased from 0° to 60°. As the widths of the trenches between the nanopillars decreased (or the diameters of the nanopillars increased) at oblique angles, the aspect ratios of the trenches increased. Incident light is likely to be more scattered in trenches with higher aspect ratios. In other words, more light was trapped in trenches with higher aspect ratios, resulting in a greater reduction in the reflectance of light. Therefore, the *R_w_* values of the Si substrates that were periodically arrayed with nanopillars decreased as the angles of their nanopillars increased.

If the increases in the aspect ratios of the trenches between the nanopillars were the sole factor for reducing the reflectance values of the Si substrates periodically arrayed with nanopillars, it could be argued that fabricating oblique nanopillars would not be necessary. In other words, the reflectance of Si substrates with vertical nanopillars could be decreased by increasing their aspect ratios instead of making them oblique.

**Figure 7:**
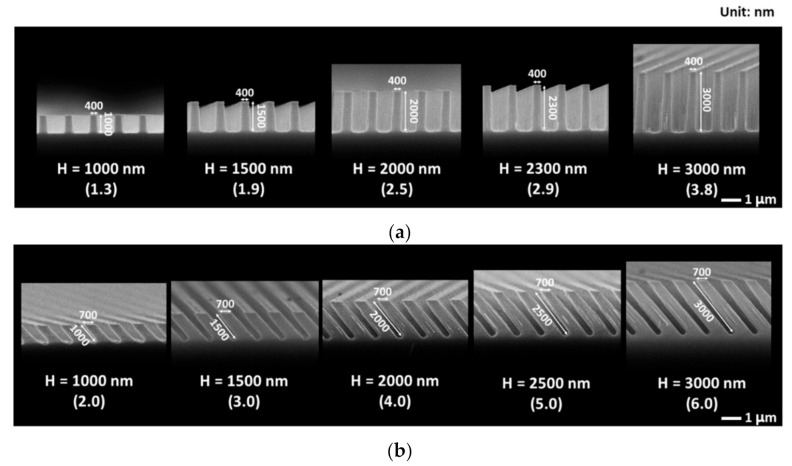
SEM images of Si substrates periodically arrayed with nanopillars at various aspect ratios: (**a**) 0°; (**b**) 40°. The numbers in the parentheses represent the aspect ratios of the trenches between the nanopillars.

To address this issue, the reflectance of Si substrates with periodically arrayed nanopillars at 0° (vertical) and 40° was compared. Figure 7 shows SEM images of Si substrates periodically arrayed with nanopillars at 0° and 40° with various aspect ratios. The diameters of the nanopillars at each angle were fixed, whereas their heights in the direction of θ were varied from 1000 to 3000 nm. By doing this, the aspect ratios of the trenches between the nanopillars at 0° and 40° were varied.

**Figure 8:**
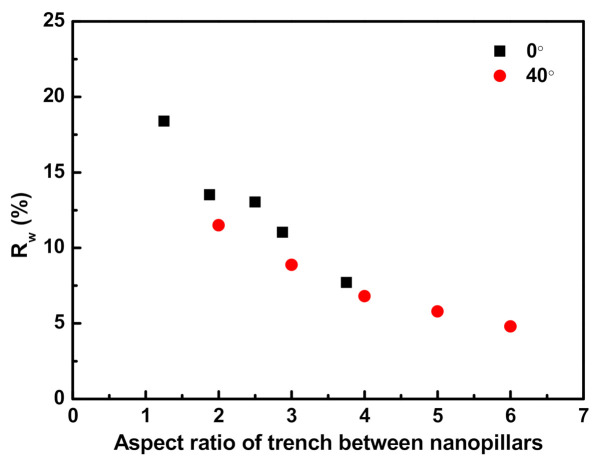
Weighted mean reflectance of Si substrates periodically arrayed with nanopillars at 0° and 40° as a function of the aspect ratios of trenches between the nanopillars.

Figure 8 shows the *R_w_* values of the Si substrates periodically arrayed with nanopillars at 0° and 40° as a function of the aspect ratios of the trenches between the nanopillars. As expected, the reflectance decreased with the increase in the aspect ratio of the trenches at both 0° and 40°. At the same aspect ratio, the *R_w_* of the trenches at 40° was lower than that at 0°.

The reduction in the reflectance of light incident on the surface of the textured Si substrate is known to result from light being trapped in the structure, as well as the change in the effective refractive index over the structure [20]. As the Fresnel reflection was located at the interface of the two media, there was a large difference in the refractive index between air and Si, leading to the reflection of light incident on the Si substrate. The nanopillars on the Si substrate served as an optical buffer, such that the effective refractive index of the nanopillar array was lower than that of Si. In addition, the tapered morphology of the nanopillar array reduced the reflection due to the gradual change in the refractive index observed over the array [21].

As seen in Figure 7, the nanopillars at 40° swelled toward the substrate compared to those at 0°. As the ion flux had a cosine distribution with respect to θ, it decreased with increasing θ. Therefore, when ions arrived off-normal to the surface of the substrate, etching was delayed compared to that for the vertical incidence of ions. As the etching process proceeded, the etching delays accumulated, resulting in the etch profiles having a tapered morphology (or the swollen structure of nanopillars) at 40°. On the other hand, when ions arrived vertically at the surface of the substrate, there was no etching delay, leading to less tapered etch profiles at 0°. Therefore, the *R_w_* of the Si substrate that was periodically arrayed with nanopillars at 40° was lower than that at with nanopillars arrayed at 0°.

## 4. Conclusions

The optical reflectance of silicon (Si) substrates with periodically arrayed nanopillars at various angles, which were fabricated via slanted plasma etching, was investigated. The weighted mean reflectance (*R_w_*) values of Si substrates with periodically arrayed nanopillars were significantly lower than that of bare Si. In addition, when the heights of the nanopillars (defined as the etch depth in the direction of the ion-incidence angle) were fixed, the *R_w_* values of the Si substrates with periodically arrayed nanopillars decreased monotonically with the increase in the angle of the nanopillars from 0° to 60°. Decreasing the width (or increasing the aspect ratio) of the trenches between the nanopillars at oblique angles resulted in more light being trapped in the trenches with the increase in the angle of the nanopillars, possibly due to the shadowing effect.

When the aspect ratios of the trenches between the nanopillars at 0° (vertical) and 40° (oblique) were the same, the *R_w_* of the Si substrate with periodically arrayed nanopillars at 40° was lower than that at 0°. This resulted from a more gradual change in the refractive index over the nanopillar array at 40° than that at 0° because the oblique ion-incidence angles of the former created a tapered morphology for the etch profiles (or because it swelled the structures of the nanopillars).

This indicates that the surface texturing of Si with oblique nanopillars is effective in reducing light reflection compared to a conventional array of vertical nanopillars.

## Data Availability

Not applicable.

## References

[1] Kim S., Kim J.-H., Kim J., Kim C.-K. (2018). Reducing the optical reflectance of kerf-loss free silicon wafers via auto-masked CF_4_/O_2_ plasma etch. ECS J. Solid State Sci. Technol..

[2] Swatowska B., Stapinski T. (2008). Amorphous hydrogenated silicon-nitride films for applications in solar cells. Vacuum.

[3] Xi J.-Q., Schubert M.F., Kim J.K., Schubert E.F., Chen M., Lin S.-Y., Liu W., Smart A. (2007). Optical thin-film materials with low refractive index for broadband elimination of Fresnel reflection. Nat. Photonics.

[4] Kim S.-K., Zhang X., Hill D.J., Song K.-Y., Park J.-S., Park H.-G., Cahoon J.-F. (2015). Doubling absorption in nanowire solar cells with dielectric shell optical antennas. Nano Lett..

[5] Pai Y.-H., Meng F.-S., Lin C.-J., Kuo H.-C., Hsu S.-H., Chang Y.-C., Lin G.-R. (2009). Aspect-ratio-dependent ultra-low reflection and luminescence of dry-etched Si nanopillars on Si substrate. Nanotechnology.

[6] Chattopadhyay S., Husng Y.F., Jen Y.J., Ganguly A., Chen K.H., Chen L.C. (2010). Anti-reflecting and photonic nanotstructures. Mater. Sci. Eng. R Rep..

[7] Lee C., Bae S.Y., Mobasser S., Manohara H. (2005). A novel silicon nanotips antrireflection surface for the micro sun sensor. Nano Lett..

[8] Zhu J., Yu Z.F., Burkhard G.F., Hsu C.M., Connor S.T., Xu Y.Q., Wang Q., McGehee M., Fan S.H., Cui Y. (2009). Optical absorption enhancement in amorphous silicon nanowire and nanocone arrays. Nano Lett..

[9] Bindra H.S., Kumeria T., Nayak R. (2018). Rapid processing of wafer-scale anti-reflecting 3D hierarchical structures on silicon and its templation. Materials.

[10] Kim S., Park J.-S., Kim J.-H., Kim C.-K., Kim J. (2019). Auto-masked surface texturing of kerf-loss free silicon wafers using hexafluoroisopropanol in a capacitivley coupled plasma etching system. ECS J. Solid State Sci. Technol..

[11] Shieh J., Lin C.H., Yang M.C. (2007). Plasma nanofabrications and antireflection applications. J. Phys. D Appl. Phys..

[12] Chen S.-H., Yeh Y.-W., Tseng S.-Z., Shih I.-T., Chan C.-H., Lee C.-C. (2012). Light harvesting analysis of a nano-cylinder structure on crystalline silicon using the Mie scattering model. J. Non-Cryst. Solids.

[13] Campbell P., Green M.A. (1987). Light trapping properties of pyramidally textured surfaces. J. Appl. Phys..

[14] Xu Z., Jiang J., Gartia M.R., Liu G.L. (2012). Monolithic integrations of slanted silicon nanostructures on 3D microstructures and their application to surface-enhanced Raman spectroscopy. J. Phys. Chem. C.

[15] Yao Y.-C., Tsai M.-T., Hsu H.-C., She L.-W., Cheng C.-M., Chen Y.-C., Wu C.-J., Lee Y.-J. (2012). Use of two-dimensional nanorod arrays with slanted ITO film to enhance optical absorption for photovoltaic applications. Opt. Express.

[16] Cho S.-W., Kim J.-H., Kang D.W., Lee K., Kim C.-K. (2014). Single- and multi-directional slanted plasma etching of silicon under practical plasma processing conditions. ECS J. Solid State Sci. Technol..

[17] Rhee H., Kwon H., Kim C.-K., Kim H.J., Yoo J., Kim Y.W. (2008). Comparison of deep silicon etching using SF_6_/C_4_F_8_ and SF_6_/C_4_F_6_ plasmas in the Bosch process. J. Vac. Sci. Technol. B.

[18] Kim J.-H., Kim C.-K. (2020). Si_3_N_4_ etch rates at various ion-incidence angles in high-density CF_4_, CHF_3_, and C_2_F_6_ plasmas. Korean J. Chem. Eng..

[19] Doshi P., Jellison G.E., Rohatgi A. (1997). Characterization and optimization of absorbing plasma-enhanced chemical vapor deposited antireflection coatings for silicon photovoltaics. Appl. Opt..

[20] Striemer C.C., Fauchet P.M. (2002). Dynamic etching of silicon for broadband antireflection applications. Appl. Phys. Lett..

[21] Jung J.-Y., Guo Z., Jee S.-W., Um H.-D., Park K.-T., Lee J.-H. (2010). A strong antireflective solar cell prepared by tapering silicon nanowires. Opt. Express.

